# Characterization of genetically effective cells and EMS mutagenesis on the novel winter oil seed pennycress (*Thlaspi arvense*)

**DOI:** 10.1093/g3journal/jkag233

**Published:** 2026-08-22

**Authors:** Anthony Brusa, Ratan Chopra, Eva Serena Gjesvold, Matthew Ott, Liam Sullivan, Claire Chapman Biel, Shweta Jain, Carl Branch, Greta Rockstad, Krishan M Rai, David Marks

**Affiliations:** Department of Agronomy and Plant Genetics, University of Minnesota Twin Cities, St Paul, MN 55108, United States; Department of Agronomy and Plant Genetics, University of Minnesota Twin Cities, St Paul, MN 55108, United States; College of Biological Sciences, University of Minnesota Twin Cities, St Paul, MN 55108, United States; Department of Agronomy and Plant Genetics, University of Minnesota Twin Cities, St Paul, MN 55108, United States; College of Biological Sciences, University of Minnesota Twin Cities, St Paul, MN 55108, United States; College of Biological Sciences, University of Minnesota Twin Cities, St Paul, MN 55108, United States; College of Biological Sciences, University of Minnesota Twin Cities, St Paul, MN 55108, United States; College of Biological Sciences, University of Minnesota Twin Cities, St Paul, MN 55108, United States; College of Biological Sciences, University of Minnesota Twin Cities, St Paul, MN 55108, United States; College of Biological Sciences, University of Minnesota Twin Cities, St Paul, MN 55108, United States; College of Biological Sciences, University of Minnesota Twin Cities, St Paul, MN 55108, United States

**Keywords:** Pennycress, Ethyl methanesulfonate, Mutagenesis, Gene index, Genetically effective cells, Intermediate crop

## Abstract

We investigated the impact of ethyl methanesulfonate (EMS) on genetically effective cells (GECs) to characterize the intraindividual genetic variation of EMS mutagenesis in the novel intermediate crop pennycress (*Thlaspi arvense* L.). We identified that pennycress contains at least four GECs which, when treated with EMS, create unique mutations across different branches within the same individual plant. However, we also found that branches were not completely distinct with 3.83% to 34.2% of the observed mutations being shared between at least two branches. We then propagated the M2 plants for whole genome sequencing, providing extensive characterization of the EMS mutation profile and developing a gene index as a resource for future reverse genetic screenings. The gene index was then validated through identification of two traits. We identified five mutations in transparent testa (*TT*) family of genes which resulted in lighter seed coat coloration and two mutations in the indehiscent (*IND*) gene which resulted in increased shatter resistance of the pennycress seedpod. These two investigations demonstrate the viability of the gene index as a resource for further reverse genetic screenings.

## Introduction

*Thlaspi arvense* L. (field pennycress or pennycress herein) collected from the wild has been targeted for domestication as a new intermediate crop that can be grown on tens of millions of acres in temperate regions during the offseason between the harvest and sowing of traditional summer crops. Importantly, this new overwintering crop provides a number of ecosystem services. As a winter cover, pennycress can reduce soil erosion, and it flowers early in the spring providing both honeybees and native pollinators with a new source of pollen and nectar. The ecosystem services of pennycress have been well characterized, including improvements in soil preservation (Cecchin et al. 2021), weed control (Johnson et al. 2015), pollinator services (Eberle et al. 2015; Evangelista et al. 2017), and water quality (Ott et al. 2019; Moore et al. 2020). Throughout its life cycle, it can also take up and utilize excess nutrients applied during the previous summer. This is especially desirable when following maize as even under the best circumstances maize only uses up to half the applied nitrogen, which can be up to 200 pounds per acre. This nitrogen typically either migrates to the ground waters, escapes in runoff polluting rivers, or is broken down into greenhouse gases. Taking up excess nitrogen pennycress provides valuable ecosystem services to both the grower and to the broader community. However, the adoption of cover crops has been slow due to the expense of sowing and terminating them in the field. Unlike cover crops, intermediate crops (such as pennycress) provide a harvestable product of value. Pennycress produces an oilseed with up to 35% oil that is suitable for conversion to biofuels, bioplastics, renewable aviation fuel, and feedstock applications of pennycress meal (Moser et al. 2009; Hojilla-Evangelista et al. 2013; Markel et al. 2018). Thus, pennycress protects soil and provides ecosystem services while also providing the farmer with a new source of income during an otherwise unproductive season.

The initial wild pennycress isolates carried both desirable and weedy traits. As a winter annual, pennycress can survive cold harsh winters while yielding over 2,000 lbs/acre of oilseeds in the mid to late spring. However, the yield can be greatly decreased by seedpod shatter during combine harvest. High levels of the glucosinolate sinigrin decrease the value of the seed meal, and the oil of wild pennycress contains high levels of toxic erucic acid. In addition, wild pennycress seeds have a thick anti-nutritional seed coat and seed dormancy that allows pennycress to create a prolonged seed bank after harvest. However, like *Arabidopsis*, pennycress is a diploid that is self-fertile. These features facilitate the use of classical chemical mutagenesis and gene editing to make homozygous recessive mutants with desirable traits. This has been demonstrated through both the screening of an ethyl methanesulfonate (EMS) mutagenized population and through gene editing to identify new varieties of pennycress that lack the undesirable or weedy traits mentioned above (Chopra et al. 2020; Marks et al. 2021; Ott et al. 2021). This work was facilitated by the advent of next generation sequencing, which was used to generate both a pennycress transcriptome and draft genome. Together these resources defined nearly all of the gene space in pennycress. These analyses also showed that, similar to the closely related *Arabidopsis*, pennycress genes are mostly present as single copies (Chopra et al. 2018; McGinn et al. 2019). This led to the prediction that in pennycress, it should be possible to isolate the same spectrum of mutants as found for *Arabidopsis* and that it should be possible to identify causative candidate gene mutations in pennycress using *Arabidopsis* as a guide.

More recently, a revised annotated genome sequence was released in 2022 (Nunn et al. 2022) and is publicly available on NCBI (https://www.ncbi.nlm.nih.gov/assembly/GCA_911865555.2). This new genome sequence contains much more comprehensive annotations than previous assemblies, allowing for better identification and characterization of lines with important agronomic traits. This sequence was based on the natural accession MN106 that was isolated from a ditch near Coates, MN. Unlike previous versions of the genome, this new version was based on MN106 after ten generations of single-seed descent to reduce natural heterozygosity.

This newly sequenced MN106 variant was used to create the EMS mutagenized population used in this work. While the chemistry of EMS mutagenesis is quite well understood, the developmental biological process via which mutagenized cells give rise to mutants in the next generation is not something that has been widely studied. In *Arabidopsis*, it is estimated that two to three genetically effective cells (GECs) in the dry seed embryo give rise to the gametes that form the next generation. During EMS mutagenesis of pre-germinated seeds, each of the GEC accumulates distinct sets of mutations that are then passed onto the next generation. In *Arabidopsis*, each GEC creates contiguous clones of cells from which seeds form on different branches or on different sides of the main branch of an individual (Irish and Sussex 1992). This phenomenon was followed up through phenotypic analyses, including Meinke and Sussex's observations on the segregation of embryo lethal mutations in siliques on sectors of the main stem (Meinke and Sussex 1979). In this case, each of the embryo lethal mutant containing sectors included one-third to one-half of the circumference of the main stem, which is consistent with the presence of two to three GECs in the seed embryo. Similar investigations in *Lotus japonicus* estimated at least three GECs were present at mutagenesis (Perry et al. 2009). To investigate if this phenomenon holds true in pennycress, we used a novel approach by performing WGS on seedlings derived from the seeds collected from separate stems of M1 individuals. This analysis provides new information on the potential number cells from mutagenized seeds that contribute to the M2 generation in pennycress, enabling investigators to better predict the possible diversity generated by EMS mutagenesis of pennycress seed.

To develop a new resource for pennycress domestication, EMS mutations in 857 M2 individuals were identified through WGS. This data was used to compile a gene index for use in reverse genetic screening. The improved annotation (Nunn et al. 2022) enabled us to identify genic non-synonymous mutations. Extensive analyses were conducted on this dataset to determine mutation numbers and rates. As a proof of concept, the data was used to perform two reverse genetic screenings, one on seed coat coloration and the other on shatter resistance. The variant information from the gene index will enable researchers to use reverse genetics to identify lines with specific mutations in a gene of interest, potentially speeding up the discovery of new agronomic traits in pennycress. To streamline this process, we developed a workflow that allows researchers to identify and filter mutants for a specific gene and obtain seed from a known, genotyped mutant pennycress plant in less than 6 months.

## Materials and methods

### Ethyl methanesulfonate mutagenesis

In order to minimize the impact of natural variation, we worked with a seed from the MN106 reference line which had been selfed through ten generations. Seed from the final generation was then treated with EMS to induce mutagenesis. Fifty thousand seeds were placed on a shaker and imbibed with distilled water overnight. These seeds were then treated with 0.2% EMS in a 0.1 M sodium phosphate buffer (pH 7.0) for 18 h. Ethyl methanesulfonate was deactivated by immersion in 0.1 M sodium thiosulfate (pH 7.3) for 20 min. Mutagenized seeds were washed in distilled water and then soaked in a 50 µM GA_4+7_ solution overnight.

Mutagenized seeds were planted in a field plot measuring 5 ft. × 50 ft. using the broadcasting method, and M1 plants were vernalized and flowered in the field. Seeds from more than 1,000 randomly selected M1 plants were harvested. Seed from the M2 individuals was planted in fall of 2020 and 2021, harvested the following spring, and pooled, providing the M3 seed which serves as the gene index long-term genetic resource.

### DNA isolation from M1 and M2 shoots and DNA sequencing

To enable the creation of a mutant gene index and to track segregation of alleles post-EMS treatment, we collected DNA from both M1 seedlings and single M2 plants. Genetically effective cell characterization was performed by collecting seeds from seven to ten unique stems/branches of M1 plants. Approximately 20 seeds from three different branches and one main stem of an M1 plant were planted on filter paper, and DNA was collected from 15-day-old seedlings using the methods described below.

To build the mutant library, M2 seeds from individual M1 plants harvested in the fields were to be planted on filter paper with water in a sandwich bag. Seeds were allowed to germinate for 7 to 10 d and then transferred to a cold chamber for vernalization. After 21 d of cold treatment, seedlings were transferred to soil in 2-in. containers for establishment and phenotyping. We grew wild-type (MN106) seeds for comparison using the same method. We collected leaf tissue from the individual which had the most robust pod fill in order to produce viable seeds for future experiments.

Approximately five young leaves from a plant of interest or ∼20 seedlings from each line of interest were collected using forceps in a 1.5 microfuge tube. These tubes were immersed in liquid nitrogen before being transferred to storage at −80 °C for later extraction. DNA was isolated using either the GenCatch TM Plant Genomic DNA Purification Kit (Epoch Life Science) or the DNEasy Plant Mini Kit (Qiagen) with some modifications to produce high-quality DNA. Briefly, frozen tissue was crushed using a microfuge tube-compatible pestle, and PX1 buffer containing RNase was to be added and homogenized by vortexing. Tubes were incubated at 65 °C for 10 min and then gently mixed after 5 min. The PX2 buffer was to be added to the tubes and incubated on ice for 15 min. After incubation, tubes were centrifuged at 15,000 rpm, and the supernatant was removed for further processing on the DNA column. The final elution (55 µl) was performed using DNase-/RNase-free water. We quantified DNA samples using nanodrop (ThermoFisher). The highest-quality samples were sent to the Joint Genome Institute for genome resequencing on an Illumina Novaseq at 15× coverage under Proposal ID: 506821.

### Bioinformatics

Sequence data of pennycress accessions were processed using a standard bioinformatics workflow. Raw reads were processed using Trimmomatic (Bolger et al. 2014) to remove low quality reads before being aligned through bwa-mem (Li and Durbin 2009). Alignment was performed against the pennycress v2.0 genome (Nunn et al. 2022). Post-alignment processing was performed using Samtools and Picard tools to remove PCR duplicates and assign read groups and for sorting. Variant calling was performed on each of the mutant aligned files separately with the GATK tool Haplotype Genotyper (McKenna et al. 2010) for both SNPs and indels. We predicted the effects of mutations in the coding regions and regulatory sequences using SNPEff (Cingolani et al. 2012). This required construction of a SNPEff database built on the v2.0 pennycress genome, and the VCF file from GATK was used as input to generate an annotated VCF file. Annotations included amino acid changes based on open reading frame, addition or removal of stop codons, variant impact, and location of the variant relative to known genes. The VCF was filtered to remove intergenic variants, and the remaining variants were exported to an Excel file for ease of use (Supplementary Table 1). One additional filtration step was performed to create a tab containing only non-synonymous variants.

### Candidate searches

Predicting the impact of known mutations is an ongoing challenge for reverse genetic screenings, and a one size fits all solution does not exist. We utilized a combination of difference resources for processing and evaluating lines and used them to identify two different examples of successful reverse genetic screenings using the MGI. Identification of candidate mutations starts with identifying genes of interest from the literature, after which the gene index can be sorted for mutations in those genes and candidate lines identified. Individual mutations from the index can then be evaluated to determine if they are worth pursuing. An example of the pipeline that we used for our work follows; candidate genes for MGI validation were identified through ongoing projects and were part of well-documented pathways, supported by literature search (Sagasser et al. 2002; Liljegren et al. 2004). All variants within these genes were isolated from the MGI and considered for evaluation. Amino acid sequence alignments against homologous *Arabidopsis* gene sequences were performed using Clustal Omega (Sievers et al. 2011), and functional annotations for the genes were obtained through UniProt (https://www.uniprot.org/) (Bateman et al. 2025) and TAIR (http://arabidopsis.org) (Rhee et al. 2003). By combining the sequence alignments with functional annotations, we were able to translate pennycress mutation positions into corresponding positions in *Arabidopsis* genes, thereby identifying mutation positions relative to functional domains. Lines with early stop codons were targeted for evaluation, as were mutations in functional regions, binding sites, or other major structural features. We also evaluated functional disruption of genes using the machine learning model PPVED (Gou et al. 2022). Candidate EMS lines contain many mutations in additional genes which many impact resulting phenotypes. We addressed this by sorting the gene index by “origin track” field which allowed us to identify all other genes which may be impacted by mutations in a given line. This information is also available through our data release at the Joint Genome Institute (ID: 506821). Ultimately, the choice of mutations to pursue is best chosen based on a combination of all available lines of evidence.

Seeds from candidate lines were germinated in a 50 µM GA_4+7_ solution. Seedlings were then vernalized for 3 weeks, transplanted into 72-well flats in a growth chamber. At the four leaf stage, leaf tissue was collected and genotyped using a competitive allele specific PCR (PACE, 3CR Bioscience) to identify homozygous mutants. These individuals were then grown to maturity, allowed to self, and evaluated for the mutant phenotype against MN106 as a control. Transparent testa mutants were phenotyped visually due to the stark difference between mutant (yellow) and wild-type (black) seed coat. Shatter resistance (*IND*) was phenotyped using a 5 N force gauge to measure the force required to break open a mature seedpod. Thirty measurements were taken per individual plant, and statistical significance was determined using comparison of means (T-test).

## Results

### Impact of GEC on EMS mutagenesis

An analysis of mutations across the main stem and three branches of individual M1 plants showed a complex combination of both shared and private mutations (Fig. 1). In the individual Sec_7, we found that 31,638 out of 42,700 mutations (73.9%) were only present in seeds collected from a single branch. The remaining mutations were present in the seeds collected from at least two branches, with only 2,981 mutations (6.9%) present in all four branches that we sequenced. Similar results were observed in the other two individuals; SEC_8 had 25,844 out of 39,791 (64.9%) mutations present only in seeds collected from single branches and 3,240 (8.14%) found in seeds collected from all branches, while SEC_10 had 19,835 mutations out of 31,751 (62.4%) present in seeds collected from single branches and 1,512 (4.76%) found in seeds collected from all branches. Across all three individuals analyzed, we found that private mutations, those found exclusively in a single branch, accounted for a majority of mutations present.

**Fig. 1.**
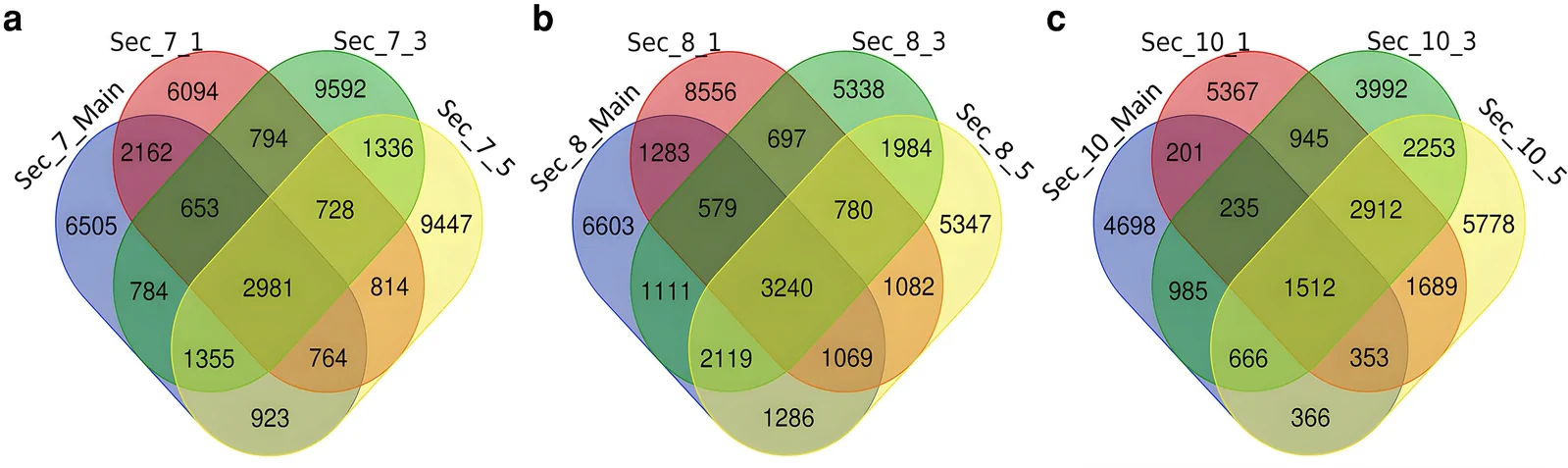
A comparison central stem and three branches show that EMS mutagenesis creates a mix shared and private mutations within branches of the same individual. Private mutations made up the majority of mutations across Sec_7 (a), Sec_8 (b), and Sec_10 (c).

Similar results were also found when sequencing M2 individuals which were derived from seed taken from differing branches of the same M1 individual (Fig. 2). Two siblings that descended from the M1 plant 201-M2-55 had 9,221 shared mutations, with the sibling plants containing 9,668 and 8,301 private mutations resulting in a roughly even split of private and shared mutations across siblings (32.7% shared, with siblings having 34.3% and 33% private). 2020-M2-142 showed a similar pattern, with 6,610 (34.2%) of mutations shared between siblings and 12,800 (46.85%) and 7,911 (29%) mutations being private. Seedlings derived from 2020-M2-933 showed a different pattern with only 282 (3.83%) mutations being shared, while the vast majority of mutations (3,938 or 53.6% and 3,127 or 42.6%) were found only in one of the siblings.

**Fig. 2.**
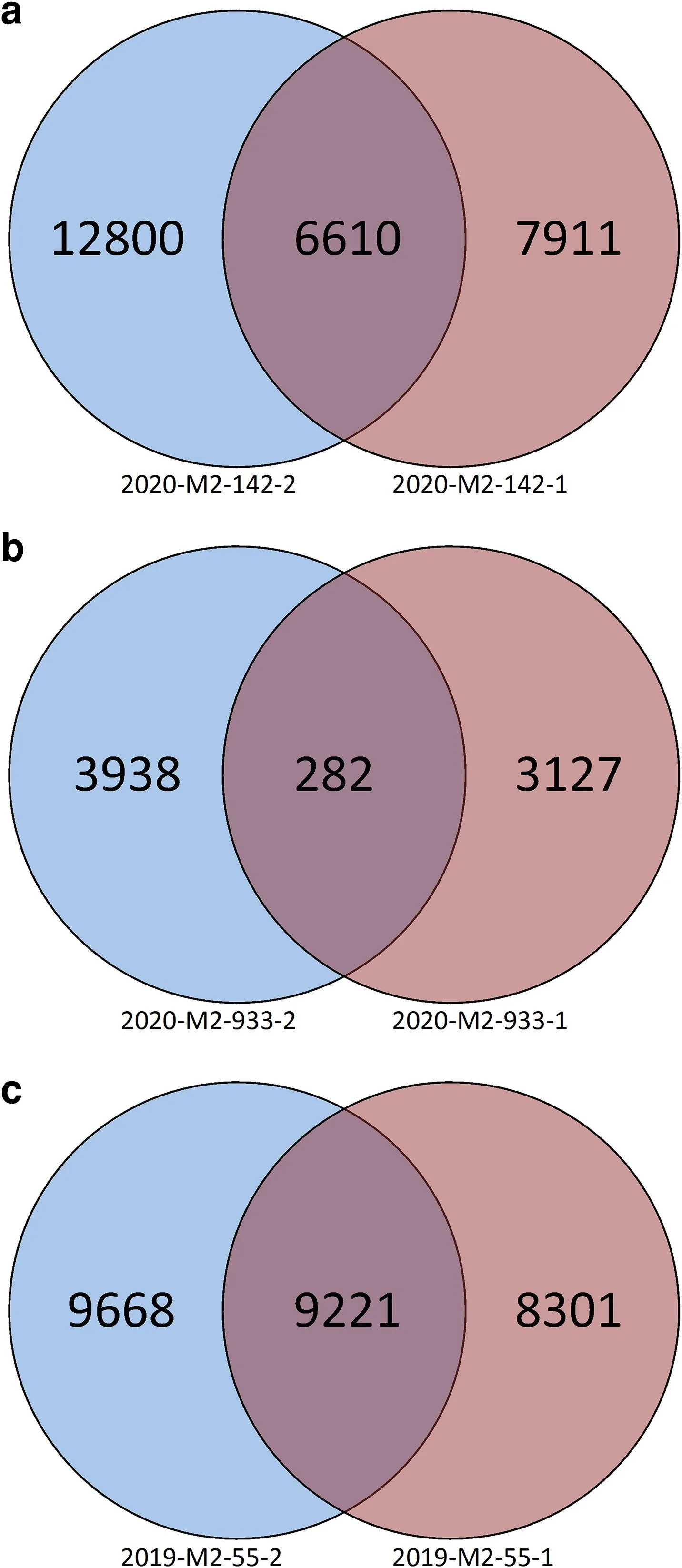
A comparison of shared and private SNPs in M2 individuals from three lines: 2020-M2-142 (a), 2020-M2-933 (b), and 2019-M2-55 (c).

These results are consistent with a limited number of GECs, but at least four, in the pennycress embryo that mostly, but not exclusively, produce contiguous clones in the aerial shoot to create the large numbers of stem-specific private mutations. However, the data is also consistent with the hypothesis that after mutagenesis and during subsequent cell divisions, there is some mixing of the original progenitors of the distinct populations of mutagenized embryonic cells. This latter phenomenon would account for the shared mutations observed between the various stems whose seeds served as the source for the WGS analyses. We put forward the hypothesis of branch progenitor cells mixing because observation of the exact same shared mutation occurring across multiple branches is unlikely to be caused by multiple independent mutations, much less so when considering the thousands of such events that would be necessary to explain the results observed in Fig. 1. This phenomenon has not previously been reported and is a departure from the results observed in *Arabidopsis*. Although beyond the scope of our work, untangling the details of this mosaicism could be done though the histological assessment of individual cells within branches. Possible approaches include the use of fluorescent probes (eg FISH) or the use of single-cell genomic sequencing.

### Characterization of M2 EMS mutations

To generate a genome-wide resource for pennycress mutants, we mutagenized a pool of MN106 (reference line) seed using EMS. From this pool of seed, we performed single-seed descent to create 857 populations of M2 plants, which we then subjected to WGS. For the final index, the nature and distribution of both genic and nongenic EMS-induced mutations were characterized, as were comparable statistics for the near-isogenic MN106 line from which EMS mutants were derived (Table 1). 3,871,244 total mutations were observed across the entire genome on the EMS lines, with 3,676,458 (95.0%) of mutations being single nucleotide variations (SNVs). MN106 was found to have 565,388 SNVs (89.4% of all mutations). Deletions and insertions represented 117,333 (3.0%) and 77,453 (2.0%) of mutations, respectively, in the EMS lines and 32,331 (5.11%) and 34,751 (5.49%) in MN106. Ethyl methanesulfonate mutagenesis preferentially causes transitions, which was reflected in a transition rate of 80.42% among SNVs compared to 33.02% in MN106. Indels represented only 5% of total variation for mutagenized lines, while indels accounted for 10.61% of mutations in MN106.

**Table 1.** Mutation overview of EMS mutagenized lines compared to MN106.

Total mutations				
Features	MGI	MN106		
Single nucleotide polymorphisms (SNPs)	3,676,458	565,388		
Deletions	117,333	32,331		
Insertions	77,453	34,751		
Total number of mutations	3,871,244	632,470		

SNP mutations			MGI % of total variation	MN106 % of total variation
A>T	54,025	30,030	1.4	5.31
T>A	57,480	30,090	1.48	5.32
C>T	1,562,272	97,278	40.36	17.21
G>A	1,551,287	97,124	40.07	17.18
A>G	98,684	93,249	2.55	16.49
T>C	98,405	93,436	2.54	16.53
G>C	24,975	14,937	0.65	2.64
C>G	25,919	15,023	0.67	2.66
A>C	35,281	23,016	0.91	4.07
C>A	66,123	24,102	1.71	4.26
T>G	36,525	23,071	0.94	4.08
G>T	65,482	24,032	1.69	4.25

Further evaluation (Table 2) showed that, among genome-wide mutations, 3,635,065 (93.9%) were found outside of genic regions and 73,213 (1.9%) variants were synonymous changes within exons. A total of 162,963 (4.2%) variants were located within exons and found to be non-synonymous, making them viable sources of novel mutations. The vast majority of these variants, 150,345 (92.2%), were missense mutations which cause single amino acid changes. Higher impact mutations were also observed: 8,160 (5%) early stop codons, 6,591 (4%) changes in splice sites, 4,084 (2.5%) frameshift mutations, and 304 (0.1%) loss of start codons. Out of 29,236 annotated genes, 88.27% had some form of unique mutation, 21.96% had a premature stop codon, and 8.69% had a frameshift mutation. Additional details, including an index of non-synonymous mutations, are available in Supplementary Table 1.

**Table 2.** Feature overview of EMS mutagenized lines.

Genic vs nongenic mutations	Number	
Non-exonic	3,635,065	
Non-synonymous	162,963	
Synonymous	73,216	
Total number of coding variations	3,871,244	
**Genic mutation types**	**Number**	
Missense	150,345	
Nonsense	8160	
Splice site	6591	
Frameshift	4084	
Start loss	304	
Stop loss	70	

INDEL size (bp)	Number	Percent of total variation
1	90,227	2.33
2	42,864	1.11
3	12,930	0.33
4	12,143	0.31
5	4209	0.11
6+	32,413	0.84

We also selected random mutant sites from the individuals in the library to make molecular markers in order to verify the presence of predicted mutations in the M3 and M4 generations. For the M3 generation, we found that 36 of 41 mutant sites tested were present in the later generations (Supplementary Table 2). For the M4 generation, we found that 24 of 30 sites tested could be confirmed as segregating (Supplementary Table 3). These results correspond to a mutation retention rate of 87.8% and 80%, respectively, demonstrating that the majority of the alleles identified at M2 generation are still present in M3 and M4 generations. However, the retention rate will be lower if lines are propagated to more advanced generations due to a variety of factors. Any real-world (non-infinite) population will lose some alleles across generations due to genetic drift, the severity of which is determined by how small the population is. Additionally, baseline segregation will result in the loss of some mutant alleles. The original M1 EMS mutations would be predominantly heterozygous; thus, we expect that a given allele in the next generation's offspring would have a one-fourth chance of being fixed and a one-fourth chance of being lost and replaced with the reference state. We addressed this potential loss of mutations for the gene index by stopping at the M3 generation and pooling seed across 200+ individuals. While this cannot completely eliminate the potential loss of mutations, the vast majority of identified mutations should be recoverable by screening enough individuals. In our experience, screening a 72-well flat of mutant seedlings has been enough to recover enough mutant alleles to work from. However, some homozygous or heterozygous mutations may also be lost due to fitness issues. Widespread EMS mutagenesis impacts tens of thousands of genes, and some of these are likely to impact a plant's fitness. These fitness issues may be caused by the alleles themselves, linked alleles, or fixed alleles elsewhere in the genome. Importantly, growing the plants in a field environment allowed only plants to be selected that are sufficiently fit to survive the challenging winter conditions of the Upper Midwestern United States. Many of the induced mutations associated with unfit plants would be lost or persist only in heterozygous individuals. While this is an important consideration for propagation of germplasm into advanced generations, the gene index itself was constructed using M2 individuals. Mutations with lethal fitness impacts would not survive for inclusion in the gene index. Additionally, the germplasm resource itself is based on bulked M3 seed. This means that any seed requested from the gene index (Supplementary Table 1) will only be one generation removed from the sequence data used to generate the index, and bulking across hundreds of plants should account for the loss of mutations in segregating individuals.

## Validation of EMS populations for use in reverse genetics

### Reverse genetic screen for pennycress seed coat mutants

The identification of transparent testa mutants with lighter seed coat color is a straightforward and easy trait to phenotype; therefore, it was selected as our initial target for reverse genetic screening. Wild-type pennycress (MN106) uniformly has a dark red/black seed coat. Lighter colors are not found in wild-type lines, but previous work has shown that that pennycress seed coat color can be altered by mutations in genes involved in the production of condensed seed coat tannins as was also observed in *Arabidopsis* (Chopra et al. 2018; Ott et al. 2021). Specifically, the disruption of the genes *TT2* and *TT8* results in a yellow seed coat, while disruption of other genes (*TT6*, *TT7*, *TT12*, *BAN*) results in an intermediate tan colored seed coat. *TT2* and *TT8* are transcription factors, while *TT6*, *TT7*, *BAN*, and *TT12* encode pathway enzymes. For this screen, sequenced lines were identified that carried a mutation in one of these genes (Table 3). Seed packets containing pooled seeds from these MGI lines were screened for the presence of lighter colors seeds, which is indicative of a defect in pathway or regulatory genes (Fig. 3). The degree of color change depends on the severity of the disruption and its position on the biosynthetic pathway, which was demonstrated in previous work (Chopra et al. 2018). Typically, strong loss of function mutations (such as nonsense or frameshift mutations) in either *TT2* or *TT8* results in the formation of yellow seeds, while partial disruptions or mutations in upstream pathway genes result in various shades of tan colored seeds. The only observed exception is a single *TT2* mutant which displayed tan seeds instead of yellow. This line had a nonsense mutation, which generally results in a complete loss of function; however, this mutation only truncated the last three amino acids of the gene. With the vast majority of the gene intact, this mutation resulted in partial loss of function instead. All of the other mutants had phenotypes similar to those previously seen for strong loss of function mutations in these genes (Table 3).

**Fig. 3.**
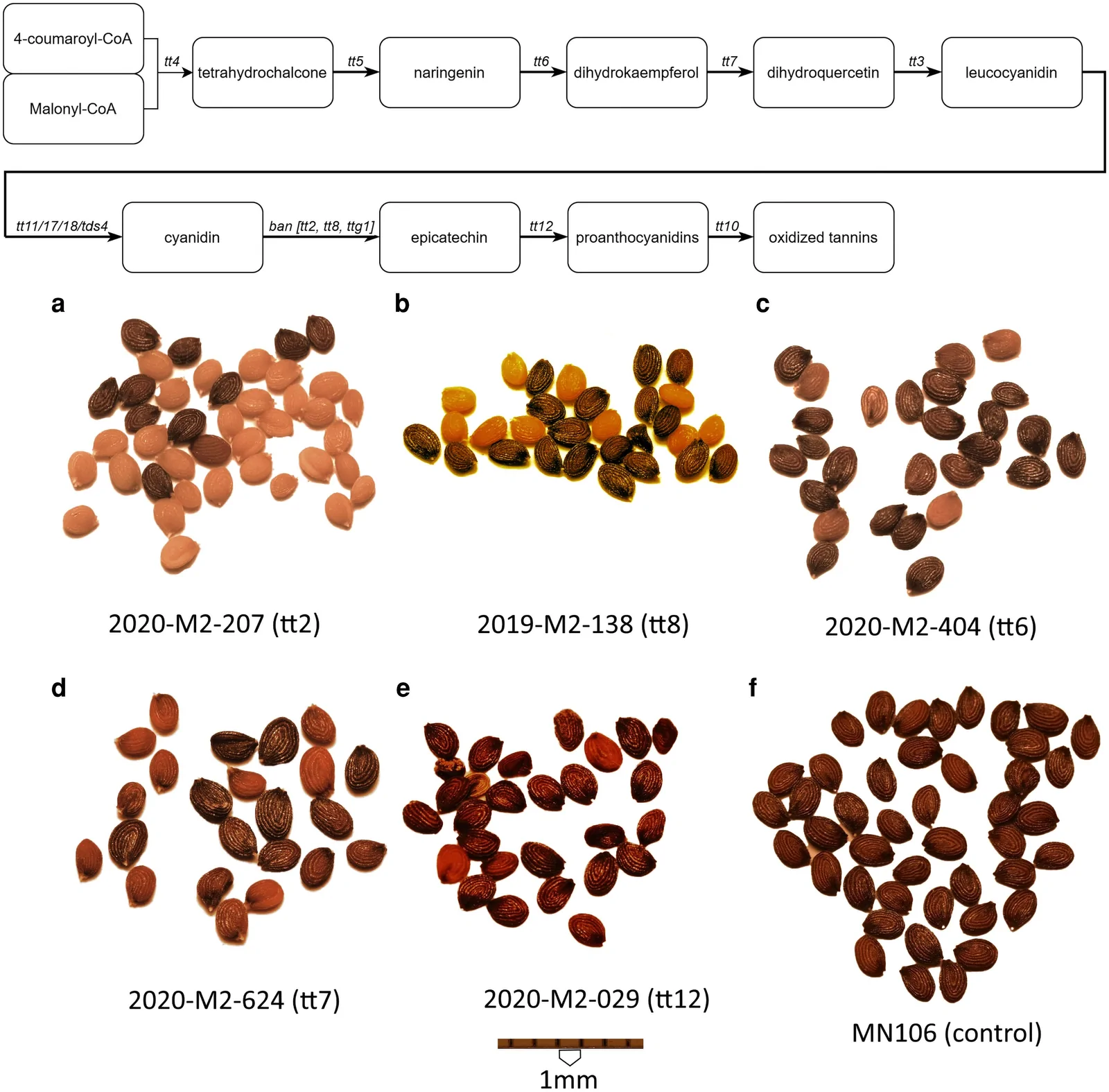
Biosynthetic pathway adapted from (Chopra et al. 2018) with images of seeds from selected pooled MGI lines. MGI lines were screened for the presence of non-synonymous mutations in genes encoding peptides required for seed coat condensed tannin formation. Shown are images from seed packets containing a mix of wild-type like and lighter colored mutant seeds. a) 2020-M2-207 *TT2*; b) 2019-M2-138 *TT8*; c) 2020-M2-404 *TT6*; d) 2020-M2-624 *TT7*; e) 2020-M2-029-*TT12*; f) MN106 parental control.

**Table 3.** Characteristics of MGI lines used in reverse screening for seed coat mutants.

Gene	Line	CHROM	POS	REF	ALT	Aa substitution	Phenotype
*TT2*	2019-M2-207	7	46787912	C	T	Arg260*	Tan
*TT2*	2020-M2-050	7	46787753	G	A	Asp207Asn	Yellow
*TT2*	2020-M2-136	7	46787409	C	T	Ser92Phe	Yellow
*TT8*	2019-M2-071	6	50472264	AAG	-	Glu230del	Yellow
*TT8*	2019-M2-138	6	50470953	C	T	Ser122Phe	Yellow
*TT8*	2019-M2-078	6	50472678	G	A	Trp317*	Yellow
*TT8*	2020-M2-832	6	50471441	G	A	Gly133Arg	Yellow
*TT6*	2020-M2-076	5	4728376	G	A	Gln325*	Tan
*TT6*	2020-M2-404	5	4728244	C	T	Ala369Thr	Tan
*TT7*	2020-M2-032	6	67293391	G	A	Thr247Ile	Tan
*TT7*	2020-M2-624	6	67292562	C	T	Trp469*	Tan
*BAN*	2020-M2-117	5	67793133	C	T	Ala182Thr	Tan
*TT12*	2020-M2-029	5	1610688	G	A	His215Tyr	Tan
*TT12*	2020-M2-865	5	1609716	C	T	Gly422Glu	Tan

Overall, 34 lines were identified carrying mutations in either regulatory or pathway genes. Within this collection, 14 lines (41%) exhibited phenotypes consistent with carrying a mutation in a gene required for normal seed coat development. Not unexpectedly, the best predictor of a phenotype was the presence of a nonsense mutation. Four of five lines with nonsense mutations displayed altered seed coat coloration. We cannot rule out the possibility that the fifth mutation might also have caused an altered seed coat, but this particular mutation was likely lost due to segregation in the generation that was used to create the mutant pool. However, this loss should be a rare event as the gene index germplasm resources were created by pooling seed from M2 plants to capture as many of the original EMS-induced mutations as possible. In any case, this screen validates the utility of the gene index population as a new source of mutants that can be identified via reverse genetic screens.

### Reverse genetic screen for seedpod shatter resistance

*IND* (indehiscent) is a gene that codes for a transcription factor involved in the differentiation of cells responsible for fruit dehiscence (Liljegren et al. 2004). It is an important gene for domesticated pennycress; disruption of *IND* results in phenotype with reduced seedpod shatter, expressed as a higher force required to spate the halves of a seedpod (Chopra et al. 2020). Four mutations in the MGI were identified within the *IND* gene, two of which demonstrated a reduced shatter phenotype (Table 4). These mutations correspond to an amino acid substitution of Gly53Asp and Leu9Phe.

**Table 4.** Characteristics of MGI lines used in reverse screening for shatter resistance.

Gene	Line	CHROM	POS	REF	ALT	Aa substitution
*IND*	2020-M2-918	6	69868	C	T	Tarvense_00017:p.Gly53Asp
*IND*	2020-M2-245	6	70001	G	A	Tarvense_00017:p.Leu9Phe

Plants were grown in a growth chamber alongside the reference line MN106. Reduced shatter trait was assessed by using a 5 N force gauge to measure the force required to open seedpods, and 30 pods were measured per individual plant. Both amino acid substitutions were accompanied by significant increase in the required force for opening seedpods (Fig. 4, *P* < 0.0001).

**Fig. 4.**
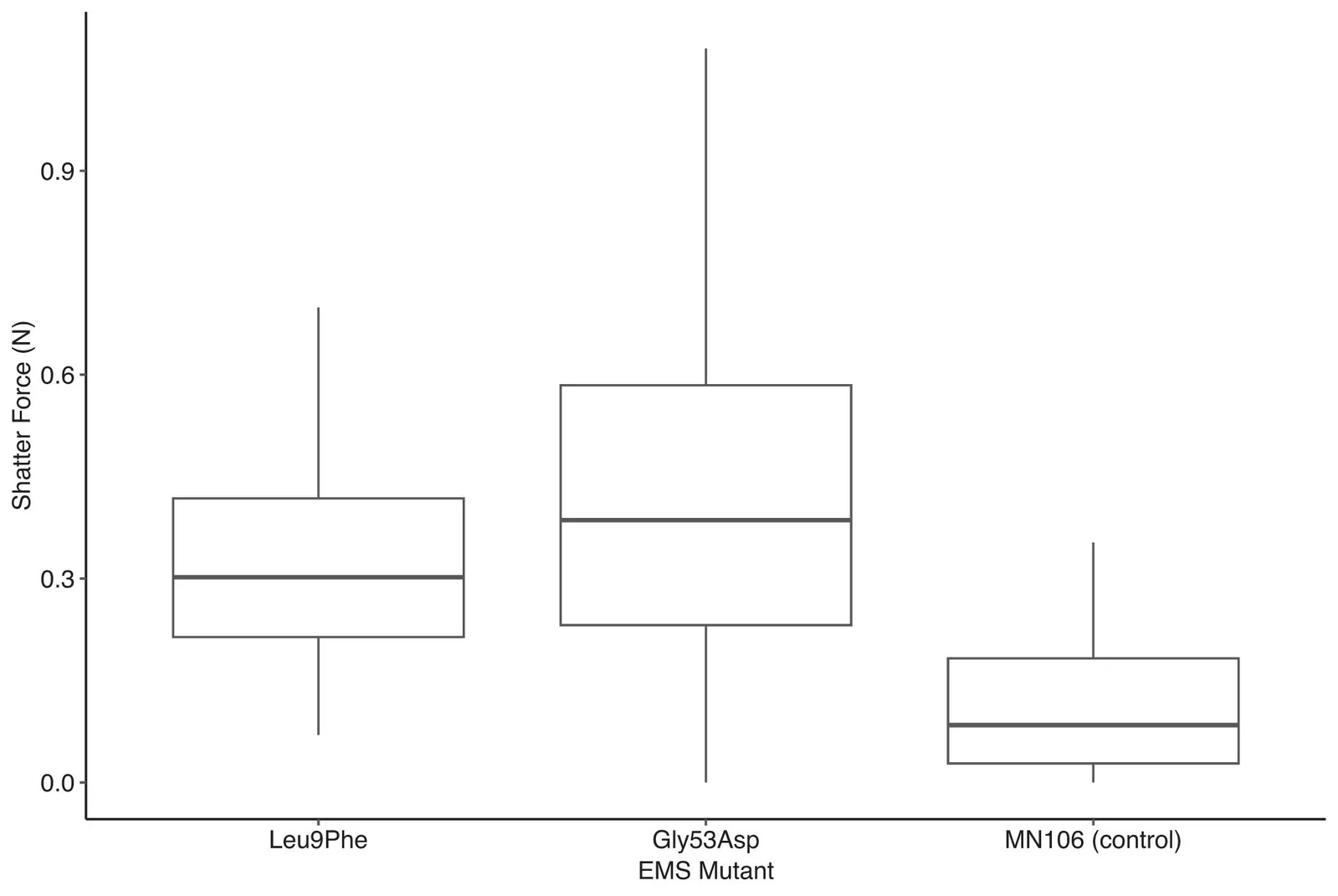
Two single amino acid substitutions (Leu9Phe and Gly53Asp) in the gene *IND* show a significant increase in force required to shatter seedpod compared to wild-type MN106.

## Discussion

### Genetically effective cell and EMS mutagenesis

Ethyl methanesulfonate mutagenesis has been widely used since the 1970s as a method of increasing genetic variation. While individual mutations are unpredictable, EMS mutation rate and preference for transitions make it more predictable than other methods such as radiation. Previous work estimated that pennycress has a background mutation rate of 7 × 10^−9^ mutations per generation per site (Geng et al. 2021) or 0.0007 mutations per Mb, while our EMS populations are estimated at 8.9 mutations per Mb. This is of the same scale as our previous work, where we observed a mutation rate of 4.09 mutations per Mb (Chopra et al. 2018). This mutation rate is slightly lower than the observed mutation rate for EMS treated *Arabidopsis* at 18 mutations per Mb (Tabata et al. 2013); however, we utilized a 0.2% EMS solution compared to a 0.5% solution in the Tabata paper. Based this work and Chopra et al. (2018), we conclude that EMS mutagenesis rate of pennycress is comparable to that of *Arabidopsis*. While studies of EMS on pennycress are limited, pennycress is highly similar to *Arabidopsis* in terms of genome size and gene copy numbers; thus, future EMS work in pennycress may consider adopting the *Arabidopsis* methodology of 0.5% EMS to obtain higher number of mutants if the increased risk of lethal mutations is considered acceptable.

According to classical plant genetics, many dicot embryonic apical meristems are composed of a two cell layer tunica in which cells propagate via anticlinal divisions to maintain the two layers as distinct entities. The larger corpus domain lies beneath the tunica. Cells in this core region undergo both periclinal and anticlinal divisions to generate the bulk of the cells. An interesting feature of most dicots, including members of the Brassicaceae, is that a small number of cells in the second layer of the embryonic tunica give rise to both female and male gametic tissue. These are the so-called GECs which form clones of cells that are maintained as distinct vertical sectors up one side of a plant or the other. Within each sector, the cloned cells contain the progenitors for both male and female gametes. This greatly simplifies the ability to conduct chemical mutagenesis on seeds from self-compatible plants, as a mutation that is created in one of the GEC is transmitted to the next generation via a classic one homozygous mutant to two heterozygous to one homozygous wild-type segregation ratio. Thus, it is possible to identify homozygous mutations in seeds that form on one of the distinct sectors of the plant.

To test this classic interpretation of plant development, M2 seeds were collected from the main stem and the three main side shoots of three different M1 plants derived from EMS mutagenized seeds. Ethyl methanesulfonate randomly creates heterozygous mutations in the diploid sets of chromosomes in the GEC. The expectation was that whole genome sequencing of seeds from different side shoots would reveal the presence of distinct pools of mutation, with the caveat that adjacent side stems would share some mutations. Further, it was predicted that seeds from that main stem would contain a unique set of mutations along with additional mutations present in each of the distinct side stems. As shown in Fig. 1, this prediction was largely satisfied. Within each dataset, four distinct pools of mutations were identified. This supports the notion that pennycress contains at least four distinct GEC in the embryonic embryo of the seed. The sharing of mutations between stems on the same plant shows that each stem was derived from more than one distinct sector of cells and thereby contains mutations derived from multiple GEC—the same for the main stem as having been derived from one main GEC but also sharing mutations with seeds derived from the side shoots. This indicates that that the progeny of the embryonic GEC creates both shared and non-shared cell populations that give rise to the next generation of male and female gametes. The discrete pools of mutations indicate the presence of sectors, but the sharing of mutations between putative M1 sectors indicates the presence of some intermingling of tissues derived from individual GEC cells. To further confirm this, we also chose three lines and sequenced two independent M2 derived from a single M1 plant of that line. We identified several unique sites in each of the M2 lines (Fig. 2) through this method. Two of these comparisons (2019-M2-55-2 and 2020-M2-142-2) showed roughly equal amounts of private and shared mutations between the siblings, although 2020-M2-933-2 showed a much stronger divergence with very few shared mutations between the two individuals observed. The cause of this difference is unknown and requires further investigation but may be related to the mosaicism observed in cells descended from GECs. The seeds grown for this comparison were randomly chosen, and 2020-M2-933 individuals may have come from seed descended from different mutation events in the GECs. The cause of this could be investigated through future work making use of larger sample size and tracking of seed origin to individual M1 branches. Despite the differences observed in our three lines, in all three cases, we were able to confirm that the initial effect of GECs conveying different of mutations across branches impacted the availability of such mutations in the following generation.

### Implications of GEC for reverse genetic screening

The results of the GEC work demonstrate that mutations induced by EMS are unevenly distributed, even within initial M1 individuals. The mosaic nature of mutation distribution means that seed from different branches can carry the alleles for drastically different phenotypes, and this impacts the availability of those alleles in downstream M2 and M3 generations. Researchers should not expect a Mendelian 1 in four homozygous mutant rate in EMS treated seed from species with multiple GECs. Based on this information, we decided that pooling seed across multiple M2 individuals from the same parent was important for the creation of the most complete mutant resource. By pooling the seed, all mutations in the gene index will be present in pooled seed, even if the rate is below that expected from Mendelian segregation. The trade-off for this is that many individuals will not have an allele for the mutation of interest, requiring a broader screening pool to obtain mutant seedlings with the desired mutation. We felt this was an appropriate trade-off for the increase coverage of more mutations due to the prevalence of reliable low cost fluorescence-based genotyping solutions such as KASP and PACE. In our validation work above, we found that a 72-well flat reliably provided enough homozygous mutants for reverse genetic screening, and the cost to isolate at least one mutant individual from a flat was approximately 15 USD. Through the use of optimal growth chamber settings and competitive fluorescence genotyping, we were able to obtain mutant seed in 4 to 5 months. Allowing for two generations of seed increase, this means that the turnaround from initial investigation to having enough mutant seed for agronomic evaluation of a trait can be completed in a single year.

### Reverse genetic screen

Previously, we reported on using a pennycress EMS population for reverse genetic mutant screen (Chopra et al. 2018). While we were able to recover a few mutants, the population in our 2018 paper presented some challenges due to the parental pennycress plants used for that work having only recently been isolated from the wild. Whole genome sequencing analyses showed that this parental population was mixed with over 131,000 natural SNPs (Dorn et al. 2015). These extra SNPs complicated the use of reverse genetics as many or most of the alleles in the population were natural and not induced by EMS. To address this, the initial wild population (designated MN106) was subjected to single-seed descent over ten generations. The progeny from the tenth generation was used to create the EMS population described in this report. The finding that over 80% of the SNPs identified in the gene index were G to A or C to T transitions favored by EMS mutagenesis compared to 34% in MN106 (Table 1) supports the notion that vast majority of the SNPs in this population were induced by the EMS treatment. Thus, this population will be superior in performing reverse genetic screens compared to the previous resource.

As a proof of concept, we were able to screen the current MGI mutant sequences to identify several classes of mutants that carry important domestication traits. These included new alleles of transparent testa mutants. These mutants have value as they typically have reduced indigestible seed coat fiber making the residual meal available after oil extraction more valuable as an animal feed supplement. In addition, these mutants typically display reduced dormancy to allow for better field establishment in the fall, and importantly, the mutations can reduce the creation of long lived pennycress seed banks that are created by wild-type dark seeds. The second class of mutations evaluated was in the *IND* gene that is required for the proper formation of the dehiscent zone in the seedpod (Liljegren et al. 2004). In wild-type plants, this zone promotes the breakage or opening of the seedpods to release the seeds. In mutants with a disruptive mutation, more force is needed to break the pods. This greatly improves yield by reducing loss due to wind induced pod breakage in the field.

While many of the MGI mutants could be recreated by new gene editing technologies, this WGS sequenced MGI population still has value as it enables discovery of rare alleles that can be crucial for agronomic traits and plant fitness. For one, the population allows ready access in a single generation to many potential mutants that would require far more time to recreate using gene editing. Second, it is likely that many desirable alleles will be partial loss of function alleles that are readily created by EMS mutagenesis but far more challenging for gene editing. For example, complete loss of function mutations in *IND* are undesirable because they result in seedpods that are highly resistant to shatter—resistant enough that when combined harvested unbroken seedpods are disposed of out the back of the machine, greatly reducing harvest yield. Partial disruptions of *IND* are favored wherein the seedpod is resistant to breakage from wind but weak enough to break open in the combine. The *IND* mutants identified in this report still show some seedpod breakage, indicating that the causative mutations are partial rather than complete loss of function mutations.

Future domestication of pennycress will involve traits obtained from a variety of sources. Traditional breeding continues to make gains in complex polygenic traits, while forward genetic screenings of both natural and EMS-induced variation are being deployed to identify the basis of new traits. Reverse genetics is an important complement to these approaches, with CRISPR allowing for precise knockout of candidate genes and optimized reverse genetics workflows enabling rapid isolation, evaluation, and seed increase of new mutant lines for downstream agronomic study.

## Data Availability

Sequence data from this project can be found at the Joint Genome Institute under Proposal ID: 506821 or at NCBI under BioProject PRJNA1239920. Sequence data for each accession in the gene index are available as separate downloads with the prefix “Thlaspi arvense 2020-M2-.” Genome annotations, including gene homologies, can be found on NCBI: https://www.ncbi.nlm.nih.gov/assembly/GCA_911865555.2. Supplemental tables are available at GSA FigShare: https://doi.org/10.25387/g3.32086224. Access to germplasm related to these resources can be obtained by contacting the corresponding author at abrusa@umn.edu. Supplemental material available at *G3* online.
